# Metabolic features of orbital adipose tissue in patients with thyroid eye disease

**DOI:** 10.3389/fendo.2023.1151757

**Published:** 2023-08-03

**Authors:** Rui Du, Fenfen Wang, Chun Yang, Jing Hu, Jiapei Liu, Qizhi Jian, Ruonan Wang, Jian Zhang, Hui Chen, Yufan Wang, Fang Zhang

**Affiliations:** ^1^ Department of Endocrinology and Metabolism, Shanghai General Hospital, Shanghai Jiao Tong University School of Medicine, Shanghai, China; ^2^ Department of Ophthalmology, Shanghai General Hospital, Shanghai Jiao Tong University School of Medicine, Shanghai, China; ^3^ National Clinical Research Center for Eye Diseases, Shanghai General Hospital, Shanghai Jiao Tong University School of Medicine, Shanghai, China

**Keywords:** thyroid eye disease, metabolic features, orbital adipose tissue, treatment targets, pathogenesis

## Abstract

**Background:**

Thyroid eye disease (TED) is the most frequent orbital disease in adults and is characterized by the accumulation of orbital adipose tissue (OAT). It can lead to eyelid retraction or even vision loss. Orbital decompression surgery serves as the primary treatment for inactive TED by removing the excess OAT. However, there is a lack of alternative treatments to surgery due to the unclear understanding of the pathogenesis, particularly the metabolic features. Accordingly, our study was implemented to explore the content and features of metabolites of OATs from TED patients.

**Method:**

The OATs used in the current study were obtained from the orbital decompression surgery of seven patients with inactive TED. We also collected control OATs from eye surgical samples of five individuals with no history of autoimmune thyroid diseases, TED, or under non-inflammatory conditions. The liquid chromatography mass spectrometer was used for the measurements of the targeted metabolites. Afterwards, we performed differential metabolite assay analysis and related pathway enrichment analysis.

**Results:**

In our study, a total of 149 metabolite profiles were detected in all participants. There were significant differences in several metabolite profiles between the TED group and the control group, mainly including uric acid, oxidized glutathione, taurine, dGMP, oxidized glutathione 2, uracil, hexose-phosphate, 1-methylnicotinamide, D-sedoheptulose 1,7-bisphosphate, and uridine 5′-monophosphate (all *p*-value < 0.05). The TED-related pathways identified included purine metabolism, beta-alanine metabolism, glutathione metabolism (*p*-values < 0.05). Our study found overlaps and differences including uric acid and uracil, which are in accordance with metabolites found in blood of patients with TED from previous study and several newly discovered metabolite by our study such as hexose-phosphate, 1-methylnicotinamide, D-sedoheptulose 1,7-bisphosphate, compared to those tested from blood, OAT, or urine samples reported in previous studies.

**Conclusion:**

The findings of our study shed light on the metabolic features of OAT in individuals with TED. These results may help identify new treatment targets for TED, providing potential avenues for developing alternative treatments beyond ophthalmic surgery.

## Introduction

As we all know, thyroid eye disease (TED) is the most common cause of orbital diseases and potentially sight-threatening ocular conditions (1). The mostly onset age of TED is ranging from 30 to 50 years (2). Clinical symptoms related to TED mainly include swelling of eyelids, watery eyes, and orbital ache. In patients with severe TED, it can also result in blindness (1).

TED is categorized as active stage characterized by inflammation and inactive stage featured by extension of orbital adipose tissue (OAT) and remodeling of connective tissues (3). Glucocorticoids are the most frequently used medication for active TED; however, recurrence might occur when glucocorticoids are deactivated. Recently, teprotumumab, a monoclonal antagonist of insulin like growth factor 1 receptor (IGF-1R), was approved for treating active TED (4, 5). For inactive TED stage in more than 60% of all the TED patients, reshaped normal orbital anatomy by removing extra OAT is prevalent, and orbital decompression surgery is currently the primary treatment (3). To develop effective TED treatments as alterations of surgery, basic research on how TED causes OAT remodeling is of great importance (6, 7).

Recently, several studies have reported that metabolic biomarkers in tears of TED patients exhibited important implication for the metabolic distinguishment of TED (8–10). Similarly, potential candidate metabolic markers for differentiating the state of TED based on plasma or urinary lipidomic analysis were also reported (11). Interestingly, Sijie Fang et al. found that glucocorticoid treatment inhibited the lineages of both Th1-cell and Th17-cell and accompanied with changes in 79 lipid profiles (12). These findings suggest that metabolites play important roles in the development and progression of TED.

Interestingly, Zhang et al. proposed that the OAT exhibits features not shown in either BAT/BRITE or WAT, suggesting important insights into adipocyte biology and the pathogenesis of OAT hyperplasia in TED as a distinctive fat depot (13). Moreover, as a metabolic organ, drugs targeting metabolic features of OAT are elusive for the lack of thorough understanding of the OAT’ metabolic features. It is worth mentioning that the metabolism in OAT and its role in the development of TED remains to be disclosed.

Importantly, metabolome technology provides novel support for the analysis of the metabolic characteristics of adipose tissue. The metabolomic analysis on tissues including OAT and visceral and subcutaneous fat could provide significant evidence for the metabolic pathogenesis and new treatment targets of TED (1, 3). Given that human adipose tissues are diverse and have heterogeneity both at tissue and organ level, better understanding of the pathogenesis of OAT may provide more potential treatment targets.

This study aimed to find out what the metabolites of OAT from inactive TED patients are like and their contents. Thus, promoting the exploration of metabolic-targeted treatment for TED would be less traumatic and effective than surgery.

## Methods

### Study participants

We have incorporated the treatment information of the seven patients with inactive thyroid eye disease (TED) into our study. Specifically, our investigation comprised seven patients with TED who underwent orbital decompression surgery due to severe proptosis at inactive stage (comprising two men and five women, with a mean age of 33.8 years) and five controls who received surgery for high myopia (comprising one man and four women, with a mean age of 39.8 years). Notably, the mean duration of TED was 5.8 years in the TED group, and no history of smoking was detected in either the TED group or the controls.

### Biological samples collection

OATs were collected from surgical samples from seven patients with inactive TED during orbital surgeries. We also collected control OATs from eye surgical samples of five individuals without any history of autoimmune thyroid diseases, TED, or under non-inflammatory conditions. OATs were extracted immediately during the ophthalmic surgery. After extraction, we weighed an appropriate amount of tissues, extracted metabolites with 80% methanol, centrifuged, aliquoted, vacuum concentrated and dried, resuspended in 120 μL of 60% acetonitrile, and took the supernatant for metabolic analysis.

### Metabolites measurement

The handling of the OAT samples is described as before. The liquid chromatography mass spectrometer selected the AB Sciex Triple Quad 6500+ System for detection of the targeted metabolites. Liquid chromatography experiment information are as follows: detection of injection volume (μL), 2; flowrate (mL/min), 0.15; column, SeQuant^®^ZIC^®^-pHILIC 5 µm; polymeric, 150×2.1mm; column temperature (°C), 45; and mobile phase, phase A of 20 mM ammonium carbonate (pH=9.0) and phase B of 100% acetonitrile.

### Data analysis

The data analysis was performed using the R software. The analyzed data from both LC and LC-MS analyses were normalized, respectively. The pathway enrichment analysis in the current study was performed using the MetaboAnalyst 3.0. The results were calculated for statistical significance using the hypergeometric test and pathway topology.

The t-test was applied to compare the metabolite levels across the TED and control groups. A non-parametric Mann–Whitney–Wilcoxon test was used for the comparison of the metabolite ratios across groups. For all analysis, a *p*-value under 0.05 was considered statistically significant in the current study.

The raw data supporting the conclusions of this article will be made available by the authors, without undue reservation.

## Results

In the current study, we explored the diverse features in the patients’ OAT metabolites with and without TED. OATs were collected from surgical samples from seven patients with inactive TED during orbital surgeries. We also collected control OATs from eye surgical samples of five individuals without any history of autoimmune thyroid diseases, TED, or under non-inflammatory conditions. In detail, our investigation comprised seven patients with TED who underwent orbital decompression surgery due to severe proptosis at inactive stage (comprising two men and five women, with a mean age of 33.8 years) and five controls who received surgery for high myopia (comprising one man and four women, with a mean age of 39.8 years). Notably, the mean duration of TED was 5.8 years in the TED group, and no history of smoking was detected in either the TED group or the controls. Furthermore, it is worth highlighting that all of the TED patients included in our study were in the inactive phase of the disease. Please refer to **Supplementary Table S1** for further details. To clarify, all participants in the TED group received anti-hyperthyroidism medication. The participants were further categorized into two subgroups based on whether they received I^131^ therapy (n=3) or not (n=4). As there was only one patient who received glucocorticoid therapy, we performed statistical analysis on patients who did or did not receive therapy for further discussion. The targeted metabolomics detection enabled the quantification of up to 149 diverse metabolic molecules, mainly consisting of carbohydrates, nucleosides, organic acids, ketones, peptides, amino acids, organic amines, aldehydes, steroids, and alkaloids.

**Figure 1:**
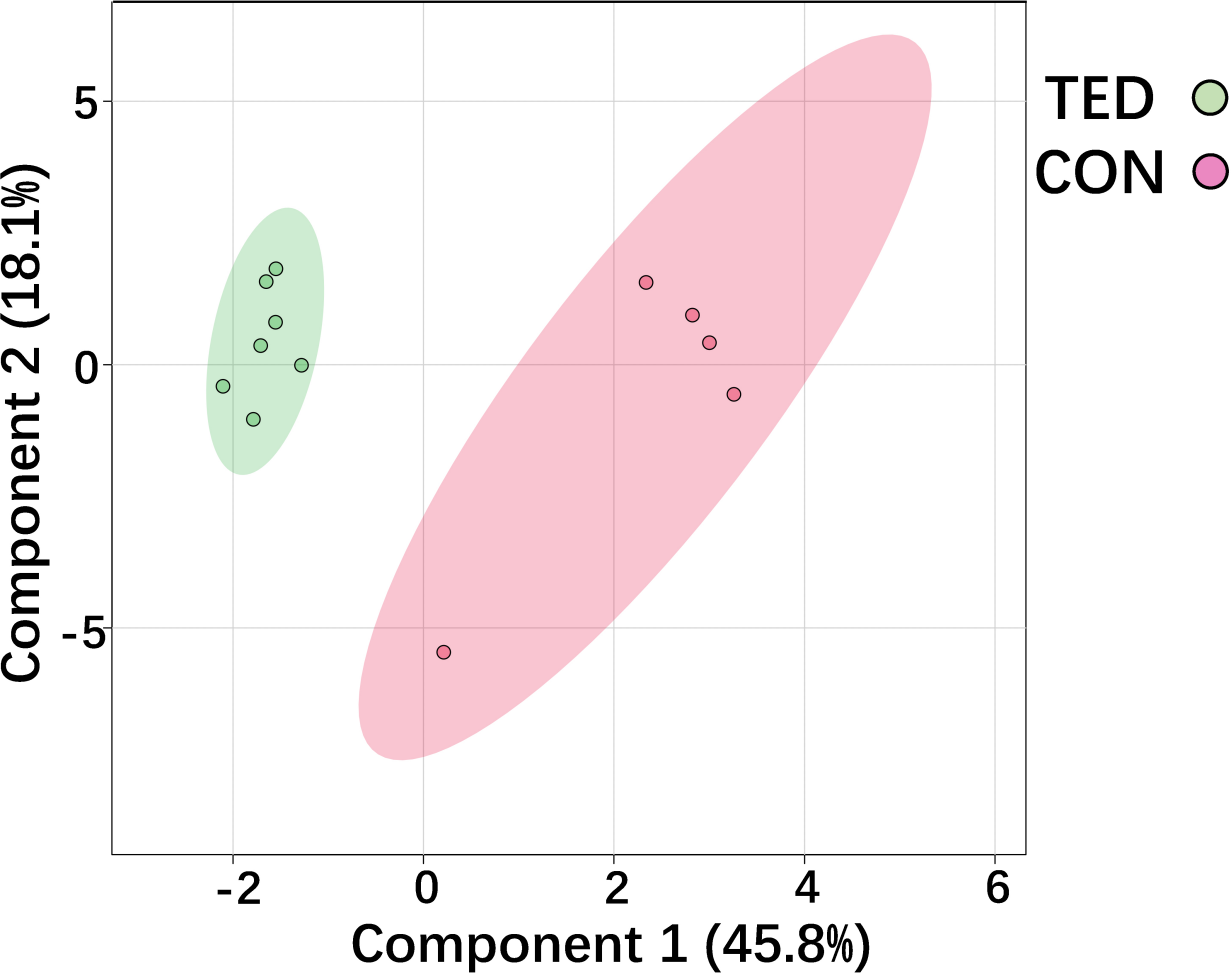
Score plot of sPLSDA model of metabolic profiles of OAT from TED patients and control.

The score plot of sparse partial least squares discriminant analysis (sPLS-DA) model indicates that TED-specific metabolic signatures differs from controls without TED (**Figure 1**). To further focus on the significantly changed metabolites in the TED group compared to the control group, we performed differential metabolite analysis. Metabolites with *p*-values <0.05 were selected. Finally, a total of 26 different metabolites were detected between TED and controls without TED (**Table 1**). The fold changes ranged from 0.23 to 13.61 (**Table 1**).

**Table 1:** Targeted metabolomics reveals the top ranks of metabolite changes in OAT from TED patients and controls.

TED vs. control		
Metabolites	Fold change	p-value
Uric acid	5.66	<0.001
Oxidized glutathione	3.41	0.001
Taurine	2.37	0.002
dGMP	1.10	0.003
Oxidized glutathione 2	2.93	0.004
Uracil	2.56	0.005
hexose-phosphate	4.87	0.008
1-Methylnicotinamide	0.54	0.008
D-Sedoheptulose 1,7-bisphosphate	8.44	0.009
Uridine 5′-monophosphate	0.44	0.009
Imidazole	0.44	0.010
AICAR	0.27	0.011
Phosphoenolpyruvic acid	13.61	0.014
N-Acetylglutamine	0.38	0.017
Betaine aldehyde	0.30	0.018
S-Adenosylhomocysteine	1.06	0.021
O-Acetylserine	0.36	0.022
Adenosine monophosphate	0.23	0.024
Argininosuccinic acid	3.58	0.024
N-Acetyl-L-alanine	2.24	0.026
IMP	0.23	0.026
Asymmetric dimethylarginine	2.32	0.028
NADH	2.30	0.035
L-Lysine	2.19	0.041
Spermine	0.37	0.042
Citicoline	2.58	0.046

In comparison with the control group, important differences in TED were detected in several metabolite profiles, such as uric acid, oxidized glutathione, taurine, dGMP, oxidized glutathione 2, uracil, hexose-phosphate, 1-methylnicotinamide, d-sedoheptulose 1,7-bisphosphate, and uridine 5′-monophosphate, respectively (all *p*-value < 0.05). Compared to TED group, there are several upregulated metabolites including uric acid, phosphoenolpyruvic acid, D-sedoheptulose 1,7-bisphosphate, hexose-phosphate, argininosuccinic acid, oxidized glutathione, oxidized glutathione 2, citicoline, uracil, taurine, asymmetric dimethylarginine, NADH, N-acetyl-L-alanine, L-lysine, dGMP, and S-adenosylhomocysteine in the control group. The phosphoenolpyruvic acid reached the most upregulation level in the TED group compared with the control group (with a fold change of 13.61). On the other hand, there exist downregulated metabolites in the TED group compared with the control group, mainly including the 1-methylnicotinamide, uridine 5′-monophosphate, imidazole, N-acetylglutamine, spermine, O-acetylserine, betaine aldehyde, AICAR, adenosine monophosphate, and IMP. The IMP reached the most downregulation level in the TED group compared with the control group (with a fold change of 0.23).

**Figure 2:**
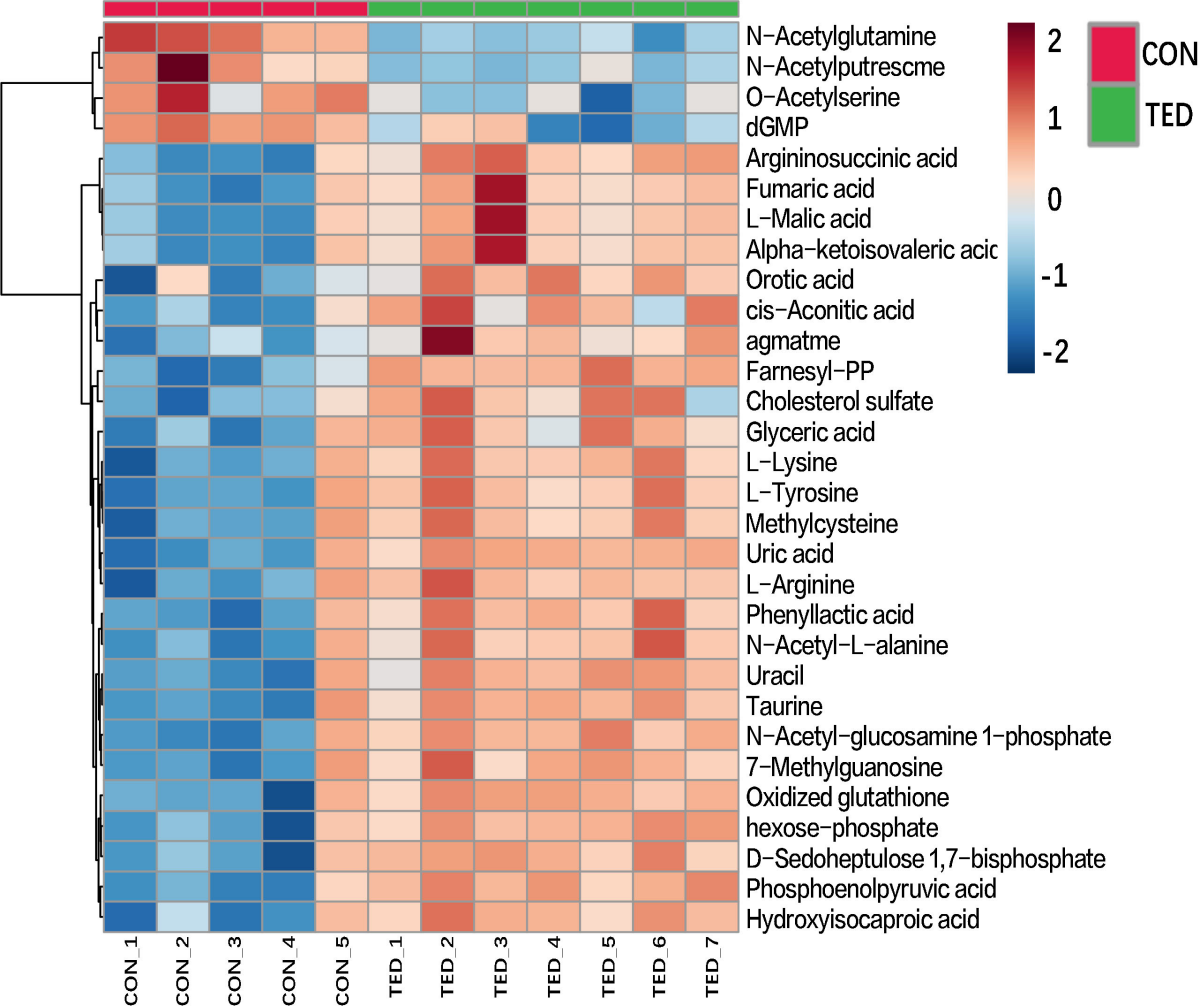
Heatmap of the top 30 different metabolites among OAT in TED patients.

In order to present more visually the results of the differential metabolite analysis, heatmaps were drawn accordingly. As is shown in **Figure 2**, the heatmap includes all the top 30 targeted metabolites, and significant differences were detected in 26 metabolites among the patients with TED and controls without TED. The upregulation is represented by the blue color patches and the downregulation by the red color patches across groups (**Figure 2**). A detailed description of the metabolites is shown in the heatmap. This heatmap is consistent with the differential metabolite analysis results described above, suggesting significant metabolite differences between groups.

**Figure 3:**
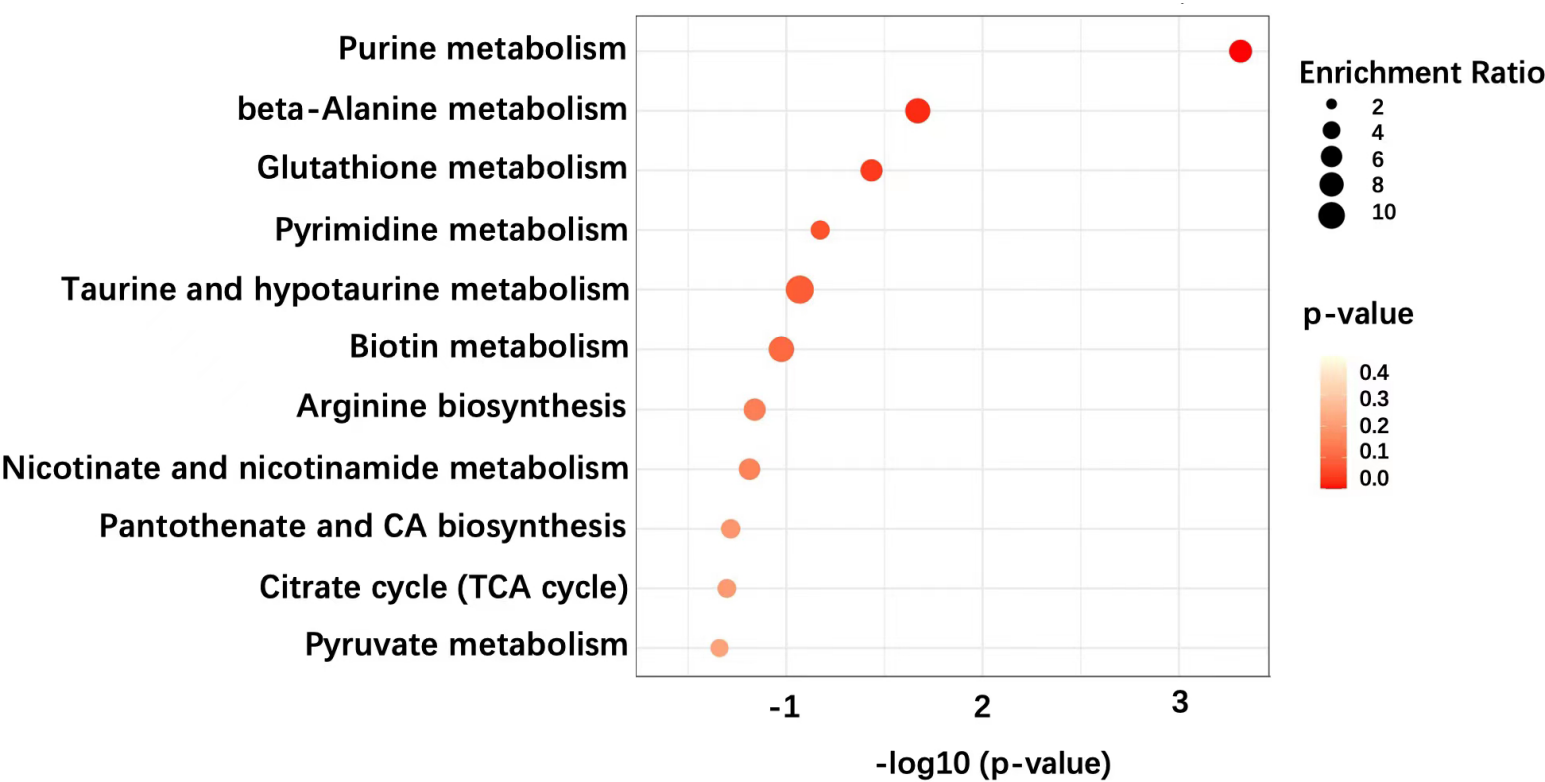
Pathway enrichment analysis of metabolic features of OAT in TED patients.

Pathway enrichment analysis demonstrated that several metabolic pathways are associated with TED (**Figure 3**). Particularly, these pathways include the purine metabolism, beta-alanine metabolism, and glutathione metabolism. These enriched pathways have overlaps and differences compared to previous studies, which is discussed in depth below. Further mechanical studies are warranted in the disclosure of these pathways’ role in the development of TED.

## Discussion

In the current study, we found 149, especially 26, metabolic differences in the OAT from TED patients compare to controls without TED. The related pathways include purine metabolism, beta-alanine metabolism, and glutathione metabolism. There are overlaps and differences among metabolic profiles tested from the OAT in our study compared with those tested from blood or urine samples reported in previous studies. Our findings shed light upon new treatment targets for TED.

According to previous studies, a majority of the metabolic analyses in TED were performed in the biological samples such as plasma and tears from patients with and without TED. Choi et al. found increased levels of malondialdehyde and 8-hydroxy-2′-deoxyquanosine in the tear samples of patients with TED (8). Moreover, Ujheli et al. showed that the concentrations of plasminogen activator inhibitor-1, IL-17A, IL-18, IL-13, and IL-6 were increased in tear samples collected from patients with TED compared with controls without TED (9). Benjamin Billiet et al. reported elevated level of spermine in the tears of patients with active TED (10). Apart from biomarkers in tears, metabolites and lipidomic profiles in the blood and the orbital tissue of patients with TED also warrants further investigation. Dong Yoon Ji et al. reported both OAT and blood metabolite profiles from TED patients (14), and a detailed comparison between our findings and their study is shown as follows. Moreover, Seul Kee Byeon et al. confirmed a different metabolism of lipid profiles associated with the pathogenesis of ophthalmopathy both in the urine and blood sample from patients with or without TED (11). The above evidence indicates that there exist both metabolic and lipidomic differences between TED patients and individuals without TED. However, the causal relationship between the reported metabolic features and TED was not fully disclosed. The analysis on metabolic features in local OAT could provide further evidence for the understanding of the pathogenesis and potential treatment targets on TED.

Our findings have overlaps and differences compared to the above-mentioned studies. In detail, a study conducted by Dong Yoon Ji et al. reported that the level of metabolic intermediates in the purine metabolism were increased in blood sample of TED patients (including IMP, xanthine, and uric acid). Our findings also confirmed that purine metabolism play an important role in the pathogenesis of TED. Similar to Dong Yoon Ji’s study, the levels of uric acid and IMP were significantly changed in the OAT of patients with TED compared to controls without TED. In our study, we did not find significant difference in the level of xanthine between groups. Moreover, Dong Yoon Ji et al. reported the upregulated metabolites in OATs consisting of fumarate, proline, and phenylalanine, which was consistent with the metabolic pattern in the blood analysis of their study. They also reported two metabolites showing significant differences between the disease groups with or without GO: 1,5-anhydroglucitol and ethanolamine. The tissue-specific metabolic dysregulation was remarked with the intermediates of nucleotide metabolism that was not identified in the blood metabolites. The abnormality in nucleotide metabolism may indicate the higher demands on RNA and DNA synthesis for proliferation under immune cell activation. However, we did not observe similar results in the metabolites from OAT. These differences might be due to diverse pathogenesis of the peripheral blood and OAT in TED. However, there is no overlap between the reported metabolites in Dong Yoon Ji’s study compared with the detected metabolites in our study. On the other hand, we found that the level of spermine was downregulated in the OAT of patients with TED rather than upregulated in tears of patients with TED reported by Benjamin Billiet’s study (10). However, in Dong Yoon Ji’s study, they did not distinguish inactive or active stage of TED in their participants. In our study, we included inactive TED patients. The above differences found in OAT’s metabolites might due to the stage of the included TED patients.

Moreover, according to findings from previous studies and the pathway enrichment analysis in our study, the arginine biosynthesis might be associated with the development of TED. Song’s study demonstrated that patients with hyperthyroidism had increased levels of ornithine and arginine compared to individuals without TED (15). Chng et al. reported that patients with TED had elevated levels of arginine when they transitioned from hyperthyroidism to euthyroidism after taking anti-thyroid drugs (16). Afterwards, Jia Liu et al. found that hyperthyroidism could significantly influence the metabolism of arginine and proline, while hypothyroidism exhibited important influence on the metabolism of alanine, aspartate, and glutamate (17). Our study also found that the level of L-arginine was elevated in the TED group compared with the controls without TED. This is the first time for the disclosure of the arginine-related metabolic pathway from the OATs in patients with TED; however, it not statistically significant. According to previously published studies, glucocorticoid therapy has been shown to modulate a lipid panel comprising 79 serum metabolites. Furthermore, in cases of glucocorticoid-therapy-resistant and very severe TED, an association has been observed between increased serum triglycerides (12). However, whether therapy has an impact on orbital metabolites remains unknown. In order to compare the characteristics of metabolites based on the differences in previous treatments for TED, we conducted a comparison of metabolite characteristics between patients who had received I^131^ therapy and those who had not. It is important to note that all the TED patients included in our study had received anti-hyperthyroidism medication. As shown in **Supplementary Table S2**, our analysis revealed that six distinct metabolites, namely, argininosuccinic acid, creatinine, L-acetylcarnitine, dimethylglycine, phosphorylcholine, and L-tryptophan, were significantly elevated in patients who had not received I^131^ therapy compared to those who had received I^131^ therapy. The above analysis suggests the differential metabolites observed in patients who have received I^131^ therapy compared to those who have not. However, it is imperative to obtain multi-center population samples to ensure the generalizability of findings. Additionally, it is crucial to observe and analyze the metabolic changes that occur at various stages of I^131^ therapy. Furthermore, further mechanistic analyses are necessary to gain a comprehensive understanding of the underlying mechanisms driving these observed differences. *In vitro* and *in vivo* mechanical studies are needed to validate the exact mechanism behind the above reported metabolic changes.

Amino acids serve as the basic components of various proteins and are significantly involved in diverse pathophysiologic processes (18). As we all know, thyroid hormones play important roles in the regulation of amino acids metabolism; as a result, there might exist opposite patterns in the amino acid metabolism under hyperthyroid and hypothyroid statuses. However, the study conducted by Jia Liu et al. reported that several amino acids showed similar level in patients with hyperthyroid and hypothyroid TED. Patients with either hyperthyroidism or hypothyroidism had elevated level of serum glutamine and downregulated level of L-glutamic acid, taurine, and L-citrulline. Our study also found that the level of taurine was increased in the TED group compared to controls without TED. These findings suggest that amino acids metabolism, especially the arginine- and taurine-related pathways might be associated in the TED development. However, these results require further investigation, and data on the metabolites in TED are limited for the present.

Adipose tissue is recognized as a highly heterogeneous endocrine organ. This heterogeneity arises from the intrinsic disparities in cellular and physiological properties exhibited by various anatomical depots. These distinctions encompass factors such as developmental origin and variances in glucose and lipid metabolism (19). The metabolic distinctions underlying the accumulation of peripheral adipose tissue in the context of obesity or type 2 diabetes have been subjected to extensive investigation (20). Boulet et al. conducted a study and observed that women with obesity had significantly higher circulating levels of branched-chain amino acids (BCAAs) and a higher kynurenine/tryptophan ratio compared to lean women or women who were overweight (21). Hanzu et al. also reported an increase in BCAA catabolism in the obesity group, along with a depletion of 2-ketoisocaproic acid in sWAT (22). In a study by Jové et al., differential expression of 31 lipids was found between sWAT and vWAT, with increased concentrations of cholesterol and cholesterol-5α,6α-epoxide in vWAT when comparing 38 subjects with obesity to lean controls (23). In addition to adipose tissues, other studies have investigated various samples from individuals with obesity and controls. Chen et al. reported on several plasma metabolites that distinguished between metabolically unhealthy obese participants and metabolically healthy obese subjects, including L-kynurenine, glycerol 1-phosphate, glycolic acid, tagatose, methyl palmitate, and uric acid (24). Furthermore, different metabolome signatures were identified between lean adolescents and those with obesity, with significantly altered levels of acylcarnitines, amino acids, glycerophospholipids, and sphingolipids in urine samples (25). As for the metabolic differences in the accumulated orbital adipose tissue in patients with TED, we found that uric acid is overlapped in our study with the study conducted by Chen et al. They reported that plasma level of uric acid is differed in metabolically unhealthy obese participants and metabolically healthy obese subjects (24). These findings suggest that orbital adipose tissue may exhibit distinct metabolic patterns compared to peripheral adipose tissue, despite both types of tissue experiencing accumulation. Interestingly, a study has observed distinct protein patterns between orbital and peripheral adipose tissue (1). In detail, Matheis et al. compared TED patients’ orbital tissue vs. peripheral subcutaneous fat. The following proteins were upregulated, namely, GTPB2 (5.6-fold), POTE ankyrin domain family member F (POTEF; 5.4-fold), kinesin family member 1A (KIF1A; 3.6-fold), lipocalin 1 (LCN1; 3.6-fold), MYH2 (3.5-fold), SPTN4 (2.9-fold), and MYH6 (2.5-fold), whereas the proteins XYLB (ratio, 0.15), REXO (0.24), and H4 (0.25) were downregulated. When comparing control orbital vs. control peripheral connective tissue, different protein patterns were observed, as described by Matheis et al.; LCN1 (3.6-fold) and GTPB2 (2.3-fold) were upregulated. However, most proteins were downregulated: XYLB (14-fold ratio, 0.07), REXO (11-fold; ratio, 0.09), PTRF (5.5-fold; ratio, 0.18), and AOC3 (3-fold; ratio, 0.35). Further investigation is necessary to gain a better understanding of the unique properties of peripheral and orbital adipose tissue.

Furthermore, genome-wide association studies (GWAS) have identified several gene associations with TED. These studies have provided valuable insights into the genetic factors underlying the development and progression of TED (26–39). One notable gene association that has been identified in GWAS is the cytotoxic T-lymphocyte-associated protein 4 (CTLA-4) gene. Variations in this gene have been found to be strongly associated with the susceptibility to TED (26). Other genes that have been implicated in GWAS include the TSHR (thyroid-stimulating hormone receptor) gene, which plays a role in the regulation of thyroid hormone production (27), and the IL23R (interleukin-23 receptor) gene, which is involved in the immune response (28). Additionally, GWAS have identified associations with genes involved in inflammation, immune regulation, and tissue remodeling, such as the PTPN22 (protein tyrosine phosphatase non-receptor 22) gene (29), the IGF1 (insulin-like growth factor 1) gene (30). Variations in these genes have been associated with an increased risk of developing TED (28). Considering the limited research on TED, we also conducted additional searches for single nucleotide polymorphism (SNP) genes associated with thyroid disorders. One of the most well-established associations is between the HLA (human leukocyte antigen) region and autoimmune thyroid diseases; particularly, the HLA-DR and HLA-DQ genes have been consistently found to be associated with an increased risk of developing autoimmune thyroid disorders (28). Additionally, TPO (thyroid peroxidase) gene, which plays a key role in thyroid hormone synthesis, have been reported to be associated with thyroid carcinoma (31) and thyroid autoimmune response (32). Furthermore, GWAS have highlighted the involvement of genes related to iodine metabolism, such as the NIS (sodium/iodide symporter) gene (33), and genes associated with thyroid hormone transport, such as the MCT8 (monocarboxylate transporter 8) gene (34).

We conducted a comparison between the genes associated with SNPs in thyroid diseases and the genes correlated with the differential metabolites and key enzymes involved in differential pathways from our study. However, we did not observe any overlap between these gene sets. This lack of overlap may be attributed to the limited research available on SNPs specifically related to TED. It is also possible that the lack of overlap between the genes associated with SNPs in thyroid diseases and the genes correlated with differential metabolites is due to variations in the expression or activity of metabolic enzymes, which can influence changes in metabolites. Compared to studies on peripheral adipose tissue, research on eye adipose tissue remains extremely limited to date. To fully understand the relationship between genetic factors, metabolic changes, and TED, future studies should investigate not only genetic variations but also the expression and activity of relevant enzymes involved in the metabolic pathways associated with the disease. This comprehensive approach will provide a more comprehensive understanding of the molecular mechanisms underlying TED and its associated metabolic alterations.

It is worth noting that the control subjects in this study were sampled from non-TED patients who had myopia, specifically from the retrobulbar adipose tissue located behind the eyeball within the orbit. This selection was made to ensure that the control group closely resembled the TED group in terms of anatomical location and tissue type. These control subjects were myopic patients who underwent surgery to remove a portion of the eyeball to achieve the restoration of ocular position due to their protruding eyes. By selecting control samples from the same location as the TED patients, we gained a comparative advantage. Recent literature reports have highlighted the structural, pathophysiological, metabolic, and molecular differences that exist in adipose tissue from different anatomical sites (40, 41). In contrast to previous studies on orbital adipose tissue, where control samples were obtained from non-retrobulbar adipose tissue in patients undergoing plastic surgery around the eyes, nose, and ears, which differs from the retrobulbar adipose tissue of TED patients (41), our choice of controls provided a more comparable location. For instance, Zhang, et al. reported that OATs had higher expression of Iroquois homeobox-family (IRX-3&5) and lower expression in HOX-family/TBX5 compared to white adipose tissue/brown adipose tissue/brown in white adipose tissue development (13). The use of controls from the same retrobulbar adipose tissue location as the TED patients enhances the validity and interpretability of our findings. It ensures that observed differences between TED patients and controls are more likely to be attributed to the disease itself rather than the variations in adipose tissue characteristics due to different anatomical locations. This study design allows for a more focused analysis of the specific effects of TED on retrobulbar adipose tissue, enabling a better understanding of the disease-related metabolic changes.

There are several limitations that should be mentioned in the current study. First, due to the tissue collection of this study, the sample size of our study is relatively small. Further large-scale or multi-center cohort studies are needed to validate these observations drawn from our study. Second, based on the observational design of the current study, we are unable to draw mechanical conclusions for the explanation of the pathogenesis in TED. Further experimental studies are needed to explore the exact mechanism behind our observations. Nevertheless, our study shed light upon the differences of metabolites in patients with TED and provides significant implications for further experimental studies concerning this issue. Third, we are not able to draw further validation among large-cohort population in the current study. Further large-scale or multi-center cohort studies are needed to validate these observations drawn from our study.

To conclude, our study identified different metabolic profiles in the OAT among individuals with TED and controls without TED. Our findings provide new insights into the treatment of TED.

## Data availability statement

The datasets presented in this article are not readily available to keep privacy of patients enrolled in the present study and in accordance with local legislation. Requests to access the datasets should be directed to zhangfang2018@sjtu.edu.cn.

## Ethics statement

The study was approved by the Ethics Committee of Shanghai General Hospital, Shanghai Jiao Tong University School of Medicine. The patients/participants provided their written informed consent to participate in this study.

## Author contributions

HC, YW, and FZ: study design and supervision. RD: data analysis, writing—original draft preparation. FW, CY, JH, JL, and QJ: writing—reviewing and editing. RW, JZ, and HC: conceptualization and methodology. All authors contributed to the article and approved the submitted version.
