# Supplementary material for: Metabolic features of orbital adipose tissue in patients with thyroid eye disease
Source: Front Endocrinol (Lausanne). 2023 Aug 3;14:1151757. doi: 10.3389/fendo.2023.1151757 (PMC10435847; doi:10.3389/fendo.2023.1151757)
Supplement: Supplementary file 1 [file Table_1.docx]

**Supplementary table 1**. Basic characteristics for the included participants.

|  | **TED**  **(n = 7)** | **Control**  **(n = 5)** |
| --- | --- | --- |
| Age, years | 33.8±8.9 | 39.8±8.1 |
| Male, n (%) | 2 (28.5%) | 1 (20%) |
| Duration of TED, years | 5.8±6.5 | 0 |
| Smoking history (yes), n (%) | 0 | 0 |
| Steroid treatment, n (%) | 1 (%) | 0 |
| Antithyroid treatment, n (%) | 7 (100%) | 0 |
| I^131^ radiation treatment, n (%) | 3 (42.8%) | 0 |
| Euthyroidism, n (%) | 2 (28.5%) | 5 (100%) |
| Hyperthyroidism, n (%) | 2 (28.5%) | 0 |
| Hypothyroidism, n (%) | 3 (42.8%) | 0 |

**Supplementary table 2.** Differential metabolites among patients with and without I^131^ radiation.

|  | **Fold change**  **(patients with/without I^131^ radiation)** | **p.value** |
| --- | --- | --- |
| Argininosuccinic acid | 0.33 | 0.004 |
| Creatinine | 0.41 | 0.026 |
| L-Acetylcarnitine | 0.61 | 0.027 |
| Dimethylglycine | 0.54 | 0.028 |
| Phosphorylcholine | 0.33 | 0.034 |
| L-Tryptophan | 0.53 | 0.036 |
